# Supplementary material for: Slowly‐adapting type II afferents contribute to conscious touch sensation in humans: Evidence from single unit intraneural microstimulation
Source: J Physiol. 2022 Jun 1;600(12):2939–52. doi: 10.1113/JP282873 (PMC9328136; doi:10.1113/JP282873)
Supplement: Supplementary file 1 — Statistical Summary Document [file TJP-600-2939-s001.docx]

**Manuscript Title:** Slowly-adapting type II afferents contribute to conscious touch sensation in humans: evidence from single unit intraneural microstimulation

**Authors:** Roger Holmes Watkins, Mario Amante, Helena Backlund Wasling, Johan Wessberg, Rochelle Ackerley

**Animal model used, if applicable:** NA

**Underlying hypothesis:** Selective SA-II afferent afferent stimulation is linked to particular tactile sensations

**Definitions of ‘n’:**

Question 1: n = number of recorded mechanoreceptive afferent neurons

Question 2: n = number of recorded mechanoreceptive afferent neurons

**Statistical summary table:**

| Experimental question number* | Finding/ conclusion | Experimental location/variable | Summary statistic | SD | n val. | P** | Units | Data comparisons | Statistical test | Any other variable | Figure/ table | Comments |
| --- | --- | --- | --- | --- | --- | --- | --- | --- | --- | --- | --- | --- |
| 1. What is the proportion of afferents that lead to tactile percepts when stimulated? | A similar proportion of SA-II afferents lead to tactile percepts when stimulated as other afferent types | Peripheral nerve | 43%  vs  44% | - | SA-II  n=14  non SA-II  n=104 | - | % afferents | SA-II vs non SA-II afferents | - | - | text | Observation  of similar proportions |
| 2. Do SA-I and SA-II perceptive fields differ in size | SA-II perceptive fields are larger | Peripheral nerve | Median  (range)  SA-II  =10 (5-30)  SA-I  =0.1 (0.01-5) | - | SA-II  n=9  SA-I  n=24 | - | Size (mm) | SA-II vs SA-I afferents | - | - | Fig 4 | Single point of overlap in data |
